# Does Kappa Agonism Improve Reversal of ‘Tranq-Dope’ Overdose? Evidence from a Rodent Model

**DOI:** 10.3390/ph19060846

**Published:** 2026-05-29

**Authors:** Michael Voronkov, Mihai Cernea, Cristina Stefanut, Georgiy Nikonov, George Milevich, John Abernethy

**Affiliations:** 1 Serodopa Therapeutics Inc., Gainesville, FL 32601, USA; gmpharmd@gmail.com (G.M.); jabernethy@serodopatherapeutics.com (J.A.); 2 Department of Pharmacology, University of Agricultural Sciences and Veterinary Medicine, 400372 Cluj-Napoca, Romania; mihai.cernea@usamvcluj.ro (M.C.); cristina.stefanut@usamvcluj.ro (C.S.); 3 Alfacheminvent LLC, Alachua, FL 32615, USA; gnikonov@alfacheminvent.com

**Keywords:** opioid use disorder, fentanyl, xylazine, overdose, polysubstance use

## Abstract

**Background/Objectives**: The recreational use of fentanyl (FT) combined with xylazine (XZ), known as “tranq-dope,” poses a growing public health threat due to its high toxicity and mortality. This study evaluated the effectiveness of naloxone (NX), its lipophilic prodrug NX90, and their combinations with the mixed κ-agonist/µ-antagonist nalbuphine (NB) in reversing overdose and restoring respiratory function in a rat model. **Methods**: Male and female Wistar rats received intramuscular FT (0.104 mg/kg) + XZ (1 mg/kg) to induce overdose, followed by intranasal administration of NX, NX90, or combinations with NB. Physiological parameters, reflex recovery, time to overdose, and reversal outcomes were assessed during individualized clinical monitoring. **Results**: At the low FT dose (0.052 mg/kg), adding XZ (1 mg/kg) shortened time to overdose by ~2600 s compared with FT alone, whereas onset times were similar at medium and high FT doses. In the dose-finding cohort, FT + XZ co-administration was associated with a higher respiratory rate than FT alone at the highest fentanyl dose tested, an exploratory finding warranting confirmation in larger studies. Most interventions did not significantly shorten time to reversal; however, NX + NB (females) and NX90 + NB (both sexes) showed shorter reversal times than NX alone. However, respiratory rate at reversal was significantly improved with NX + NB, ½NX90 + NB and NX90 + NB (90 ± 6, 86 ± 5 and 92 ± 5 breaths/min) compared with naloxone alone (80 ± 6 breaths/min). Interventions containing nalbuphine (κ-agonist/µ-antagonist) yielded higher RR and HR at reversal than NX alone, consistent with an interpretive framework in which κ–µ opioid balance may influence observed physiological recovery patterns. **Conclusions**: Comparable or improved reversal outcomes could be achieved using half-doses of NX or NX90 with NB—potentially reducing the total dose of naloxone and mitigating the risk of precipitated withdrawal in individuals with opioid use disorder.

## 1. Introduction

The opioid epidemic is a major public health crisis that has been exacerbated by the recent widespread availability of fentanyl and the adulteration of illicit drugs with dangerous additives. In 2024, provisional national data indicate that approximately 79,384 drug overdose deaths occurred in the United States, representing a substantial decline from prior years, while synthetic opioids—primarily illicitly manufactured fentanyl—remained the leading drivers of opioid-related fatalities [1]. Fentanyl causes opioid-induced respiratory depression (OIRD), which, without prompt intervention, can lead to hypoxic brain injury, long-term disability [2,3], or even death. Since fentanyl became prevalent in the U.S. drug supply, there has also been a dramatic rise in non-fatal opioid-related overdose, reaching 69.6 per 100,000 population in 2022 [4]. Contributing to the severity of the crisis is the emergence of xylazine—an α2-adrenergic receptor agonist known to cause respiratory depression in humans [5,6]—as a frequent additive in the opioid supply. The rise in xylazine prevalence has coincided with a flattening of the non-fatal overdose curve and a sharp increase in fatal overdoses, suggesting that fentanyl–xylazine (FT-XZ) combinations may produce more lethal effects. One explanation is that characteristics of fentanyl OIRD in the presence of xylazine have changed and became deadlier. Indeed, when ingested by humans, xylazine alone induces a respiratory depression of its own [5,6], as well as dangerously low blood pressure, reduced heart rate (HR), and potentially death [7]. Furthermore, recent studies linked xylazine-adulterated fentanyl to NX-resistant OIRD in humans [8,9]. Additionally, emergent animal data have confirmed the synergistic effects of fentanyl with high doses (3–32 mg/kg) of xylazine on overdose [10].

On the other hand, the pharmacological interactions between FT and XZ might be more nuanced depending on their doses and ratios. For example, Kiyatkin showed that while a low dose of FT (0.02 mg/kg) dramatically reduces brain oxygenation by about 12–15 µM at the onset of overdose, the presence of XZ (1 mg/kg) seemed to blunt this effect to only about a 7–9 µM reduction at the onset of overdose [11]. Others reported that xylazine reduces the µ-agonistic effects of FT depending on the ratio of the two drugs [12]. Finally, the recently reported k-agonism of xylazine [13] in view of its reported alleviation of µ-opioid respiratory depressant effects in rats [14,15] may also play a role in data heterogenicity.

As is quite often the case in polysubstance use, the interpretation of overdose data with FT and XZ should be based on their pharmacokinetic profiles, which requires extensive modeling [16]. Therefore, besides FT and XZ doses and ratios, timing of overdose onset may become an important factor. One would expect the impact of concentrations of each drug in the brain during an early-onset overdose to differ significantly from that in a delayed-onset scenario, potentially leading to distinct pharmacodynamic outcomes.

With respect to FT-XZ overdose reversal, the complicated pharmacology is likely playing a role in conflicting reports on the efficacy of NX [8,9,17,18] or NX in combination with α2-adrenergic receptor antagonists. NX with atipamezole was effective in reversing FT-XZ overdose when a low fentanyl dose (0.02 mg/kg) was used in rats [19]. However, at a more typical fentanyl dose (0.1 mg/kg), another α2-adrenergic receptor antagonist yohimbine alone or in combination with naloxone failed to reverse fentanyl–xylazine respiratory depression [17]. If dose, ratio, and timing determine ‘tranq-dope’ outcomes, can any single regimen provide broadly effective reversal across clinically relevant scenarios?

First, it remains unclear whether any single agent, at any dose, can effectively reverse a polysubstance overdose that engages multiple pharmacological pathways. However, several promising agents are currently under development to address fentanyl overdose, which may serve as a starting point for a “one-catches-all” intervention approach. These include: (a) mAb [20,21] and other fentanyl sequestrants [22], (b) fentanyl-based antagonists [23,24], (c) improvements in existing µ-antagonists [25,26]), (d) an emerging class of various naloxone potentiators [27,28], and (e) NX release formulations using nanoparticles [29] or triggered by hypoxia [30].

Second, prior findings have demonstrated that a “one-catches-all” intervention may be feasible by stimulating respiratory function. Specifically, a selective antagonist of large-conductance calcium-activated potassium (BKCa) channels normalized blood gases at a low dose of FT (0.02 mg/kg) in the presence of XZ (3 mg/kg) in rats [31]. This approach is agnostic to the multiple pharmacological pathways causing respiratory depression and does not rely on countering each driver of the respiratory depression separately.

Given that κ-opioid receptor agonists have also been shown to stimulate respiration, we explored a third approach—countering OIRD specifically while simultaneously stimulating respiratory function in the FT + XZ overdose model in rats. In this study, we report the respiratory rate (RR) and heart rate (HR) effects of low, medium, and high doses of fentanyl, with and without xylazine, as well as the efficacy of NX, NX90, and their combinations with k-agonist/µ-antagonist nalbuphine.

## 2. Results

**Figure 1:**
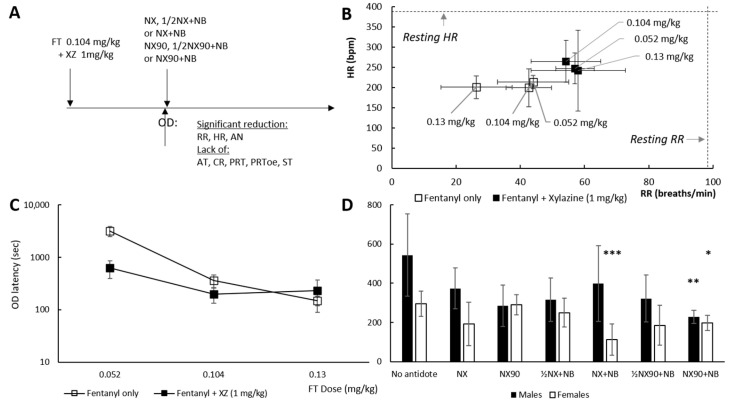
Experimental design, dose-finding, and overdose characteristics in the fentanyl–xylazine model. (**A**) Schematic of the experimental timeline and monitoring protocol for respiratory rate (RR), heart rate (HR), and six standard reflexes (AN—alertness, AT—astasia, CR—corneal reflex, PRT—pinch reflex tail, PRToe—pinch reflex toe, and ST—sternal recumbency) used to define overdose onset and recovery. (**B**) RR (breaths/min) and HR (beats/min) at overdose onset in FT alone versus FT combined with xylazine (XZ, 1 mg/kg) at low, medium, and high FT doses. XZ co-administration modified FT-induced respiratory and cardiovascular depression at overdose onset; (*n* = 4). (**C**) Dose–response relationship of overdose latency in the presence of 1 mg/kg XZ (*n* = 4). (**D**). Time to recovery of all five reflexes (s) after overdose in rats treated with equimolar doses of NX, its lipophilic prodrug NX90, or their combinations with nalbuphine: ½NX + NB (0.10 + 0.10 mg/kg), NX + NB (0.20 + 0.10 mg/kg), ½NX90 + NB (0.13 + 0.10 mg/kg), and NX90 + NB (0.26 + 0.10 mg/kg). * *p* < 0.05, ** *p* < 0.01, *** *p* < 0.005; Statistical comparisons were performed using one-way ANOVA with Bonferroni-adjusted post hoc tests. Significance is reported relative to the no-antidote control group (*n* = 5/sex for treatment groups; *n* = 7 for control groups). Data are presented as mean ± SEM. For the dose-finding studies (panels (**B**,**C**)), *n* = 4 rats per group (2 males, 2 females). For the main reversal study (panel (**D**)), *n* = 5 males and 5 females per treatment group, as described in Section 4.

The overall experimental timeline and monitored endpoints (RR, HR, reflexes) are summarized in Figure 1A.

### 2.1. Dose Selection

#### 2.1.1. XZ Dose Selection

We defined the onset of the overdose based on the following criteria: (i) all five standard reflexes are completely inhibited; (ii) the rat no longer responds to any painful, tactile, or acoustic stimuli; and (iii) there is at least a 33% decrease in RR and HR, compared to the resting rates, with overdose onset recorded as the first time point at which all criteria were simultaneously met. To select the XZ dose, we tested 0.13, 0.39, 1, 3, and 5 mg/kg administered alone. At low doses (0.13 and 0.39 mg/kg) of XZ, the most affected reflex was alertness, especially in females. At higher doses (1, 3, and 5 mg/kg), XZ produced a progressive overdose phenotype with average onset times of 1500, 645, and 458 s, respectively, and with the full recovery taking more than 60 min. At overdose onset, RR was depressed across doses (59 ± 12, 66 ± 16, and 57 ± 8 breaths/min), as was HR (222 ± 42, 229 ± 16, and 241 ± 18 beats/min). Animals at 3 mg/kg and all animals at 5 mg/kg doses required the administration of atipamezole to survive. The reported values reflect combined data (two females + two males per group; total *n* = 4). Considering the potentially synergistic effects of combining of XZ with FT, we decided that doses higher than 1 mg/kg could unjustifiably increase the risk of death for the animals. Therefore, we set the standard reference dose for XZ at 1 mg/kg, IM.

#### 2.1.2. FT Dose Selection

For this study, we also evaluated low, medium, and high FT doses at 0.05, 0.104, or 0.13 mg/kg, which correspond to a 0.6–1.5 mg human dose—just under the 2 mg lethal dose [32]—and are likely to be more representative of the fentanyl contents in street opioids. With FT alone, overdose onset occurred at ~3200 s (low), 360 s (medium), and 150 s (high), as shown on Figure 1C. The reported values reflect combined data (two females + two males per group; total *n* = 4). The high FT dose also required intervention with NX90 at 0.26 mg/kg in most animals to prevent cardiopulmonary arrest. Therefore, we selected an FT + XZ combination of 0.104 and 1 mg/kg, respectively.

### 2.2. FT + XZ Overdose Onset

With FT + XZ (1 mg/kg), overdose onset was 630 ± 232 s (low FT), 169 ± 67 s (medium), and 230 ± 141 s (high) (*n* = 4 per dose level). These values indicate a flattened dose–response relationship for onset time in the presence of xylazine, in contrast to the clear dose-dependent decrease in onset time observed with fentanyl alone.

### 2.3. RR and HR at Overdose Onset

As shown in Figure 1B, RR and HR at the time of overdose onset decreased in a dose-dependent manner with fentanyl alone, falling to approximately 40–70 breaths/min and 200–260 beats/min across the low, medium, and high FT doses, whereas the addition of xylazine (1 mg/kg) modulated the fentanyl-induced bradycardia and respiratory depression across all fentanyl doses, increasing RR by roughly 15–20 breaths/min and HR by 20–40 beats/min at each dose. At the highest FT dose, RR was higher in the FT + XZ group than FT alone (2.2-fold difference in this small exploratory cohort). Although sample size was limited, the observed effect was directionally consistent across animals.

### 2.4. Overdose Reversal

Overdose reversal was defined as full restoration of all five reflexes (100%) with corresponding improvements in RR and HR. We found that time to reversal without antidote was 543 ± 210 s in males and 296 ± 65 s in females. Time to reversal was not consistently altered by the interventions, with the exceptions of NX + NB in females (113 ± 80 s) and NX90 + NB in both sexes (198 ± 38 s in females; 228 ± 33 s in males), as shown on Figure 1D.

**Figure 2:**
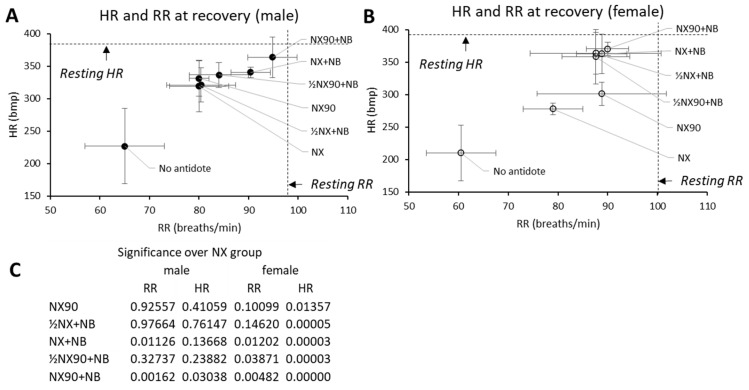
Effects of naloxone, NX90, and nalbuphine combinations on respiratory and cardiovascular recovery after fentanyl–xylazine overdose. (**A**) Respiratory rate (RR, breaths/min) and heart rate (HR, beats/min) at the time of overdose reversal in male rats treated with NX (0.20 mg/kg), NX90 (0.26 mg/kg), or their combinations with nalbuphine: ½NX + NB (0.10 + 0.10 mg/kg), NX + NB (0.20 + 0.10 mg/kg), ½NX90 + NB (0.13 + 0.10 mg/kg), and NX90 + NB (0.26 + 0.10 mg/kg). Active treatment groups: *n* = 5; no-antidote and NX control groups: *n* = 7. (**B**) RR and HR at overdose reversal in female rats (*n* = 5) for the same treatment groups. Active treatment groups: *n* = 5; no-antidote and NX control groups: *n* = 7. (**C**) Summary of statistical significance for the effects of each intervention on RR and HR at reversal relative to the NX-only group, indicating which combinations significantly improved respiratory and/or cardiovascular recovery compared with NX. Data are presented as mean ± SEM. For all treatment groups, *n* = 5 males and 5 females, as detailed in Section 4. Statistical comparisons were performed using one-way ANOVA with appropriate post hoc multiple comparisons versus the NX-treated group.

RR and HR at overdose reversal: The corresponding values for all other interventions and the negative (no antidote) and positive NX (0.2 mg/kg) controls are summarized in Table 1 and illustrated in Figure 2A,B.

### 2.5. WCS-like Phenotype

In the presence of XZ, we observed a fentanyl-associated rigidity state—marked by chest and abdominal stiffening, limb extension or spasms, transient apnea, and SpO_2_ decline—that we used as a behavioral and clinical proxy to model Wooden Chest Syndrome (WCS) in rats. At low FT doses, this WCS-like rigidity developed gradually (approximately 20 min post-administration) and could persist for up to 40 min, whereas at medium and high FT doses, onset was rapid (within about 2 min) but resolved more quickly, typically within 8–10 min.

## 3. Discussion

### 3.1. Idiosyncrasies of “Tranq-Dope” Overdose

#### 3.1.1. Lack of Dose Response in Overdose Onset

Unlike FT alone, the FT + XZ combination (“tranq-dope”) showed a plateauing dose–response curve with respect to overdose onset (Figure 1C). Specifically, RR and HR at overdose onset were largely unaffected by further escalation of FT dose, whereas FT alone produced a clear dose-dependent suppression of RR (Figure 1B). This pattern suggests that in the presence of xylazine, the fentanyl dose–onset relationship is no longer strictly monotonic, reflecting a context-dependent reweighting of drug contributions to the endpoint.

#### 3.1.2. Sex-Specific BW Effect on Overdose Onset

In females, body weight was positively associated with overdose onset time within a subgroup of animals corresponding to the upper half (above median) of the body weight distribution in the full cohort. This subgroup was used to assess the relationship between body weight and onset time without resuscitation confounding and was not selected based on the correlation outcome. A strong positive association was observed (*n* = 11, Pearson r = 0.864, R^2^ = 0.75, *p* < 0.0001) indicating that heavier females tended to reach overdose more slowly. However, this correlation was not observed in males or in FT-only overdoses. This may indicate that, in some females, FT exposure at the time of overdose was relatively lower and XZ contributed more strongly, but we did not directly measure FT/XZ levels to confirm this. Additionally, previously published pharmacokinetic data on FT + XZ showed no difference in T_max_ [33] but showed that both AUC_∞_ and C_max_ for FT were lower in the presence of XZ, suggesting relatively reduced fentanyl exposure and a greater xylazine contribution. With the observed longer time to overdose onset in females, their overdose phenotype may reflect a context-dependent shift toward xylazine-dominant over fentanyl-dominant pharmacological effects. Consistent with this, females demonstrated shorter reversal times across all groups (Figure 1D) and generally improved RR and HR recovery profiles (Figure 2C). However, this does not imply that xylazine-dominant toxicity at higher doses (>1 mg/kg) is inherently less severe, as α2-mediated cardiovascular effects may independently influence the clinical course. Although the observed inter-animal variability was larger than we have previously reported in this FT overdose model [26], a reduction in the mean time of overdose reversal was observed across all interventions, reaching statistical significance in the NX + NB- (females) and NX90 + NB-treated (both sexes) groups.

#### 3.1.3. Wooden Chest Syndrome-like Rigidity

In the presence of XZ, we observed a fentanyl-associated rigidity state that we used as a behavioral proxy for Wooden Chest Syndrome (WCS) in rats, characterized by chest and abdominal stiffening, limb extension or spasms, transient apnea, and SpO_2_ decline. This WCS-like phenotype showed a clear dose-dependent temporal pattern: at low FT doses, rigidity developed gradually (≈20 min post-administration) and could persist for up to 40 min, whereas at medium and high FT doses, onset was rapid (within ≈2 min) but resolved more quickly, typically within 8–10 min. These observations were recorded as part of the continuous behavioral monitoring framework of the study. Quantitative characterization (including incidence, duration, and SpO_2_ metrics) is not reported here, as WCS-specific analyses are reserved for a dedicated follow-up study. Given that xylazine is a potent α_2_-adrenergic agonist, these rigidity patterns likely reflect dynamic shifts in α_2_-adrenergic tone superimposed on µ-opioid receptor activation, which may help explain why “tranq-dope” overdose can present with prolonged chest stiffness for some dose combinations and shorter, more abrupt WCS-like episodes for others.

#### 3.1.4. Respiratory Rate at the “Tranq-Dope” Overdose

Most surprisingly, in the dose-selection study (*n* = 4/dose), adding XZ (1 mg/kg) was associated with higher RR at overdose onset across ascending FT doses (Figure 1B). These data suggest that xylazine may alter the pattern of fentanyl-induced respiratory depression, potentially shifting from slower bradypnea toward a different respiratory phenotype. While initial dose-finding comparisons were limited in size, they showed a directionally consistent pattern in which xylazine was associated with a modified overdose phenotype rather than simply intensifying classic fentanyl-induced bradypnea. This pattern is consistent with the plateauing dose–response pattern observed in the onset of “tranq-dope” overdose and parallels prior reports in which xylazine-modified fentanyl-induced brain oxygenation changes at overdose onset [11]. While this is compatible with xylazine’s reported weak κ-opioid receptor agonist activity—which has been shown to counteract some μ-opioid respiratory depressant effects in rats [14,15]—we interpret it primarily as a modulation of the temporal profile of respiratory depression at the doses and endpoints studied here, rather than as evidence of a protective effect on overall respiratory or cardiovascular risk. It is also consistent with reports describing higher opioid dose tolerance in patients exposed to xylazine prior to fatal overdose [34]. These findings contrast with the previously reported synergistic respiratory depression of much higher xylazine doses (3–32 mg/kg) combined with fentanyl, reinforcing the conclusion that polysubstance overdose outcomes are highly dependent on the specific doses and ratios of the drugs involved. These seemingly paradoxical findings can be reconciled by recognizing that FT + XZ interactions are highly dependent on dose, ratio, route, and the respiratory endpoint chosen. More specifically, these findings suggest that the respiratory phenotype of “tranq-dope” overdose reflects a shift in the predominant pharmacologic influence, where reduced fentanyl exposure and increased α_2_-adrenergic contribution from xylazine alter the pattern of respiratory depression without reducing overall toxicity, in parallel with the WCS-like rigidity patterns observed in our model, and consistent with FT-XZ pharmacokinetic data and the dose- and sex-dependent idiosyncrasies of “tranq-dope” overdose outlined above.

### 3.2. Quality of the Reversal

While the time to overdose reversal was not significantly influenced by the type of reversal agent used, the quality of the reversal varied substantially (Figure 2A,B, Table 1).

NX (0.20 mg/kg) served as the positive control and clinical standard-of-care comparator, so improvements in RR and HR at reversal were interpreted relative to NX rather than solely against the no-antidote group. Thus, in the NX-treated males, at the time of reversal, both RR and HR—the most critical physiological parameters—modestly recovered to 80 ± 6 breaths/min and 319 ± 40 beats/min, respectively. In contrast, NX + NB- and NX90 + NB-treated males showed significant improvements in respiratory function over NX controls (Figure 2A). Similarly, in the NX-treated females, at the time of reversal, both RR and HR recovered to 79 ± 6 breaths/min and 278 ± 9 beats/min, respectively. In contrast, regimens containing NB or NX90 significantly improved HR in all other interventions, while NX + NB and NX90 + NB (including the half-dose NX90 combinations)-treated females showed significant improvements in respiratory function over NX controls. Any supplementation with κ-agonism seemed to significantly improve HR (Figure 2B). Overall, we found that RR in males and HR in females were more sensitive to the type of the reversal agent. This sex difference in pharmacodynamic outcomes perhaps could be explained by females having a later onset of overdose that is consistent with a more “tranq”-like overdose.

Notably, the combination with the highest expected k-agonism (NX90 + NB) produced the most complete recovery of respiratory function in both sexes, coming closest to baseline levels. This suggests a more comprehensive physiological reversal of overdose compared to other treatment combinations. These findings support our hypothesis that effective reversal of “tranq-dope” overdose may require both μ-opioid receptor antagonism to counteract OIRD and k-agonism to stimulate respiratory function.

### 3.3. K-Agonism Effect on Respiratory Function and Heart Rate

To build on our observation that κ-active regimens improved “tranq-dope” reversal, we qualitatively ordered the treatments by expected μ-receptor antagonism and κ-opioid receptor agonism. For μ-antagonism, we anticipated the following hierarchy: NX + NB > NX > ½NX + NB > NX90 + NB > ½NX90 + NB > NX90, reflecting greater μ-block with full versus half-doses of naloxone or NX90 and with their combinations. For κ-agonism, we expected NX90 + NB > ½NX90 + NB > ½NX + NB > NX + NB > NX90 > NX, consistent with nalbuphine providing the strongest κ-receptor activation in the panel, NX90 displaying weaker κ-agonism, and naloxone acting as κ-antagonist [26,35]. This dose-based ordering assumes that half-doses engage fewer receptors than full doses and is used only as a qualitative framework to interpret the RR and HR distributions in Figure 2, rather than as a quantitative measure of “net” μ- or κ-activity. In line with this qualitative ordering, Figure 2A,B shows that the regimens with greater expected κ-receptor contribution—particularly NX90 + NB and ½NX90 + NB—cluster closest to resting RR and HR at the time of overdose reversal in both males and females, whereas NX alone and NX90 alone remain further from baseline despite also achieving formal reversal. Consistent with this framework, NX + NB and NX90 + NB produced the largest and most consistent improvements in RR and HR at reversal relative to NX alone, and several of these differences reached statistical significance (Figure 2C), supporting an association between regiments enriched in κ-agonism and better physiological quality of reversal.

This finding aligns with previous literature [15,16] and supports our hypothesis that supplementing μ-antagonism with κ-agonism may significantly improve respiratory function in “tranq-dope” overdose. Interestingly, our data suggest that the previously reported reversal of fentanyl-induced respiratory depression by nalbuphine [36] was likely more nuanced than previously thought and also could be attributed to its κ-agonist properties. The observed improvements in heart rate also align with prior studies documenting the cardioprotective effects of nalbuphine in fentanyl-anesthetized individuals [37,38]. Within this model, κ-agonist ranking was associated with greater physiological improvement, which we interpret as an association rather than confirmation of receptor-level causality.

## 4. Materials and Methods

### 4.1. Animals

A total of 146 *Rattus norvegicus*—Wistar breed, males and females (non-pregnant), aged 4–6 months and with body weights of 163–430 g (average body weight: 278 g)—were used. The test groups were homogeneous in terms of body weight and male-to-female ratio. The rats were purchased from the accredited Laboratory Animals Unit of the Experimental Medicine Center, University of Medicine and Pharmacy, Cluj-Napoca, Romania. Rats were housed in groups of 2–3 for one week in a climate-controlled facility (22 °C with approximately 60% relative humidity), under a 12 h light/dark cycle, with food and water available ad libitum.

All procedures in the study complied with the guidelines of Directive 63/2010/EU and National Law 43/2014 on the protection of animals used for scientific purposes. The project was carried out with the approval of the Bioethics and Research Ethics Committee of the University of Agricultural Sciences and Veterinary Medicine Cluj Napoca (435/13 March 2024), and the project authorization was issued by the National Sanitary Veterinary and Food Safety Authority (406/29 April 2024). The animals were accommodated and used for the experiments within the project at the Unit for Breeding and Use of Laboratory Animals of the University of Agricultural Sciences and Veterinary Medicine Cluj Napoca, which operates on the basis of the Veterinary authorization 926/6 June 2021. The ALURES code for the research is: NTS-RO-406074 v.1, 8 October 2025.

### 4.2. Chemicals, Experimental Groups, and Protocol

#### 4.2.1. Xylazine Overdose Model—Determination of Optimal Dose

To determine the optimal xylazine dose for inducing overdose, a single ascending dose study was performed in 20 rats. Xylazine (Xilazin Bio 2%, 20 mg/mL, Bioveta a.s., Ivanovice na Hané, Czech Republic) was administered at five doses (0.13, 0.39, 1, 3, and 5 mg/kg) to separate groups (2 males and 2 females per dose).

#### 4.2.2. Fentanyl and Xylazine Combination Overdose Model

This phase of the study aimed to establish the optimal Fentanyl dose to induce overdose when combined with xylazine (1 mg/kg). Fentanyl (Fentanyl Kalceks, 500 µg/10 mL, Kalcex, Riga, Latvia) was administered at three doses (0.052, 0.104, and 0.130 mg/kg) to separate groups (2 males and 2 females per dose). For confirmatory purposes, additional rats were tested in groups where overdose was consistently observed: the 0.104 mg/kg group (dose selected for the main study) was expanded with 6 additional males and 6 females; the 0.130 mg/kg group received 1 additional male and 1 female.

#### 4.2.3. Overdose Reversal Agents Used

Naloxone (Forvel, 0.4 mg/mL, producer Medochemie, Limassol, Cyprus), nalbuphine (Mallinckrodt Pharmaceuticals, Hazelwood, MO, USA), and NX90 (synthesized and characterized as previously described by Alfacheminvent LLC, Alachua, FL, USA powder was dissolved in sterile saline solution 0.9%. Yohimbine (MilliporeSigma, Burlington, MA, USA) was used only as an emergency adjunct to NX90 in cases of cardiac arrest during overdose procedures and was not part of any experimental treatment condition or statistical analysis in this study.

#### 4.2.4. Main Study Protocol

The main study (Figure 1A) included six active treatment groups, each consisting of 5 males and 5 females, receiving NX (0.20 mg/kg), NX90 (0.26 mg/kg), ½NX + NB (0.10 + 0.10 mg/kg), NX + NB (0.20 + 0.10 mg/kg), ½NX90 + NB (0.13 + 0.10 mg/kg), or NX90 + NB (0.26 + 0.10 mg/kg). In addition, the positive control NX group and the negative control ‘no-antidote’ group each consisted of 7 males and 7 females, providing larger reference cohorts for between-group comparisons. Each rat underwent individualized clinical monitoring for heart rate (HR), respiratory rate (RR), oxygen saturation (SpO_2_), rectal temperature (RT), and blood pressure (BP), using validated equipment: HR and RR were measured with the IM8 VET System (Cardiacdirect, San Diego, CA, USA); and SpO_2_, RT, and BP with the M3T Vet Monitor (Tootoo Meditech Co., Ltd., Shenzhen, China).

Five standard reflexes were assessed: alertness (AN): response to acoustic and brief painful stimuli (dorsal skin pinch); astasia (AT): presence of locomotor imbalance or lack of movement despite stimulation; corneal reflex (CR): eyelid closure in response to corneal stimulation with a sterile cotton applicator; righting reflex (RRef): ability to right itself when placed in a supine position; sternal recumbency (SR): ability to rise from a sternal position when supported on all four limbs.

Additionally, clinical signs of Wooden Chest Syndrome (WCS) were monitored, including chest and abdominal stiffness, limb spasms, apnea, rigid limb extension, and SpO_2_ decrease. A WCS-like episode was operationally defined as the presence of at least two of these signs occurring simultaneously and persisting for ≥10 s.

Measurements were taken following intramuscular administration (hind limb) of the fentanyl (0.104 mg/kg) and xylazine (1 mg/kg) combination as follows. Rats were monitored every 2 min during the first 10 min, and every 10 min thereafter, up to 60 min. Time to overdose and recovery were recorded at these intervals; if either occurred between checks, timing was assigned at the point of observation. Additionally, two more measurements were taken before treatment (ATp) 10 min prior to drug administration and post-treatment (PTp) 60 min after drug administration.

All animals were monitored simultaneously by two trained members of the research team, with results confirmed by a supervising investigator. Each reflex was tested three times at each time point. Reflex presence was scored as 100%, absence as 0%. The researchers responsible for reflex assessment and physiological monitoring were blinded to the treatment administered.

Upon confirming overdose (all 5 standard reflexes are completely inhibited; the rat no longer responds to painful, tactile, or acoustic stimuli; there is at least a 33% decrease in respiratory rate (RR) and heart rate (HR) compared to ATp0), reversal agents were administered intranasally (IN) within 5–10 s, depending on group assignment, body weight, and antagonist concentration. Intranasal (IN) administration was used for reversal agents to model a rapid, non-invasive, and potentially clinically translatable route of delivery, whereas intramuscular (IM) administration was used for fentanyl–xylazine induction to ensure consistent and reproducible systemic exposure. Following our previous experience with fentanyl overdose reversal in rats, we chose a 0.2 mg/kg dose for NX and an equimolar 0.26 mg/kg dose for NX90 [26], as well as 0.10 mg/kg for NB [15]. The following overdose agents and their combinations were tested: 1. NX (0.20 mg/kg); 2. NX90 (0.26 mg/kg); 3. ½NX + NB (0.10 + 0.10 mg/kg); 4. NX + NB (0.20 + 0.10 mg/kg); 5. ½NX90 + NB (0.13 + 0.10 mg/kg); 6. NX90 + NB (0.26 + 0.10 mg/kg).

Reversal time was defined as the point at which all five reflexes had returned, the rats displayed normal mobility and responded to stimuli (painful, tactile, and acoustic), and the RR and HR at that time point were recorded as indices of the physiological quality of reversal. Between-group differences in RR and HR at reversal were subsequently compared with the NX (0.20 mg/kg) group, with statistically significant deviations were interpreted as improvements or impairments in cardiorespiratory recovery.

### 4.3. Statistical Analysis

Data were analyzed using repeated-measures ANOVA (factors: time and treatment) in GraphPad InStat 3.10. Post hoc multiple comparisons were performed using Bonferroni correction following significant omnibus effects. Various statistical tests were used, with significance expressed via *p*-values. Sex-based comparisons were assessed with an unpaired *t*-test with Welch’s correction. These comparisons were performed independently of the repeated-measures ANOVA framework. For multiple comparisons, treatment effects were evaluated within defined ANOVA families using Bonferroni-adjusted post hoc tests, with NX serving as the reference condition. Comparisons were performed within each endpoint and stratified by sex where applicable. Statistical significance was defined as *p* < 0.05. Where multiple comparisons were performed, adjusted significance thresholds were applied as specified above. Correlation analysis was conducted on animals without resuscitation confounding to avoid intervention-related bias in onset timing.

### 4.4. Study Limitations

While the best effort was made to keep all cohorts and sexes at the same age, the animals used in the initial dose selection were on average younger than the animals used in the main study. With the observed protective body weight correlation in females, this could inadvertently lead to a slightly different nature of the “tranq-dope” overdose, at least in some animals. NB alone was not tested in this study since it did not have a significant improvement in RR and HR in fentanyl overdose [15].

## 5. Conclusions

Our data suggest that “tranq-dope” overdose, at fentanyl doses representative of street levels, can differ from fentanyl-only overdose in several key ways: (i) overdose onset time was not dose-dependent; (ii) higher fentanyl doses were associated with faster resolution of WCS; (iii) female subjects tended to show slower overdose onset, quicker reversal, and more favorable respiratory rate and heart rate outcomes than males.

This study provides further evidence that the outcomes of polysubstance overdose depend on specific doses, drug ratios, and the speed of overdose onset and supports a model in which “tranq-dope” overdose reflects a dynamic shift in the balance between µ-opioid and α_2_-adrenergic mechanisms, with xylazine potentially contributing an increasingly prominent α_2_-adrenergic component at certain dose and ratio combinations rather than a simple additive µ-opioid effect. We therefore interpret our data as consistent with a shift in the relative contributions of µ-opioid versus α_2_-adrenergic mechanisms across the FT:XZ parameter space, with xylazine becoming more functionally dominant under some conditions without implying any intrinsic reduction in total drug burden. Some phenotype differences, including the respiratory rate findings observed in the dose-finding cohort, should be considered exploratory and warrant confirmation in larger-powered studies. These findings should be interpreted as preclinical, non-comparative evidence of pharmacodynamic modulation rather than evidence of regimen superiority.

Within this model, we demonstrate the feasibility of an approach that counters OIRD while simultaneously supporting respiratory function through regimens that combine µ-receptor antagonism with κ-agonist/µ-antagonist nalbuphine. While most tested interventions showed no significant difference in reversal time, the presence of a low dose of a k-agonist/µ-antagonist nalbuphine significantly improved RR and HR outcomes at the time of the reversal compared with naloxone alone.

These findings may have potential clinical relevance, as nalbuphine is commercially available and has a favorable safety profile. Unlike other k-agonists, it produces minimal euphoria, respiratory depression, or neuropsychiatric side effects such as dysphoria or hallucinations—though mild sedation, nausea, and dizziness may occur [39,40].

Moreover, similar or improved physiological recovery was observed using reduced doses of naloxone or its lipophilic prodrug NX90 in combination with nalbuphine, suggesting context-dependent changes in antagonist requirement under κ-modulated conditions within this model. These observations require independent validation in non-sponsored datasets.

## Figures and Tables

**Table 1:** Physiological values at overdose reversal and nadir oxygen saturation.

Antidote	Group			Females			Males		
	RR (Breath/min)	HR (Beat/min)	Nadir SpO_2_ (%)	RR (Breath/min)	HR (Beat/min)	Nadir SpO_2_ (%)	RR (Breath/min)	HR (Beat/min)	Nadir SpO_2_ (%)
No antidote	63 ± 8	218 ± 50	89.5 ± 4.6	61 ± 7	210 ± 43	92.0 ± 0.8	65 ± 8	227 ± 58	87.0 ± 5.7
NX	80 ± 6	297 ± 32	91.1 ± 3.1	79 ± 6	278 ± 9	92.4 ± 1.7	80 ± 6	316 ± 34	89.8 ± 3.8
NX90	84 ± 10	316 ± 27	92.1 ± 3.8	89 ± 13	301 ± 18	92.6 ± 4.2	80 ± 2	331 ± 27	91.6 ± 3.9
NX + NB	90 ± 6	352 ± 33	88.4 ± 2.4	89 ± 13	363 ± 32	87.6 ± 2.4	90 ± 7	341 ± 26	89.2 ± 2.4
½NX + NB	84 ± 6	343 ± 34	92.7 ± 3.7	88 ± 5	364 ± 30	93.0 ± 4.5	80 ± 4	321 ± 8	92.4 ± 3.2
½NX90 + NB	86 ± 5	348 ± 24	90.6 ± 3.1	88 ± 7	359 ± 42	90.2 ± 4.0	84 ± 6	337 ± 19	91.0 ± 2.2
NX90 + NB	92 ± 5	367 ± 22	91.9 ± 2.4	90 ± 4	370 ± 11	91.6 ± 2.5	95 ± 5	364 ± 31	92.2 ± 2.6

## Data Availability

The raw data supporting the conclusions of this article will be made available by the authors on request.

## Abbreviations

The following abbreviations are used in this manuscript:

FT	Fentanyl
HR	Heart rate
NB	Nalbuphine
NX	Naloxone
OIRD	Opioid-induced respiratory depression
RR	Respiratory rate
XZ	Xylazine
